# *Trichoderma asperellum* (T42) and *Pseudomonas fluorescens* (OKC)-Enhances Resistance of Pea against *Erysiphe pisi* through Enhanced ROS Generation and Lignifications

**DOI:** 10.3389/fmicb.2017.00306

**Published:** 2017-03-02

**Authors:** Jai S. Patel, Ravindra N. Kharwar, Harikesh B. Singh, Ram S. Upadhyay, Birinchi K. Sarma

**Affiliations:** ^1^Department of Botany, Banaras Hindu UniversityVaranasi, India; ^2^Department of Mycology and Plant Pathology, Institute of Agricultural Sciences, Banaras Hindu UniversityVaranasi, India

**Keywords:** pea, powdery mildew, monolignol, ABC transporter, MAP kinase, NADHP oxidase

## Abstract

Plant signaling mechanisms are not completely understood in plant–fungal biotrophic pathogen interactions. Further how such interactions are influenced by compatible rhizosphere microbes are also not well-studied. Therefore, we explored the pea-*Erysiphe pisi* (obligate biotroph) system to understand the interaction and applied compatible rhizospheric bio-agents *Trichoderma asperellum* (T42) and *Pseudomonas fluorescens* (OKC) singly or in combination to assess their influence on the host while under the pathogen challenge. Transcript accumulation pattern of some vital genes in the lignin biosynthetic pathway in pea under *E. pisi* challenge indicated enhanced activation of the pathway. Interestingly, transcript accumulations were even higher in the bio-agent treated plants compared to untreated plants after pathogen inoculation particularly in co-inoculated treatments. Further, down regulation of the lignifications-associated ABC transporter gene in the pathogen challenged plants possibly is an indication of passive diffusion of monolignols across the membrane from symplast. Additionally, up regulation of NADPH oxidase gene revealed ROS generation in the challenged plants which was confirmed through spectrophotometric estimation of H_2_O_2_. Up regulation of laccase and peroxidase along with higher H_2_O_2_ generation points out their involvement in lignifications which was further confirmed through cross section analysis of pea stems that showed increased lignifications in pathogen challenged plants co-inoculated with the bioagents. Interestingly, pathogen responsive MAPK homologs MAPK3/MAPK6 and the enzyme serine threonine kinase that activates MAPKs were down regulated and the results possibly indicate non-participation of the MAPK cascade in this interaction. Therefore, it can be concluded that the microbial treatments enhanced pea resistance to *E. pisi* by generation of ROS and lignifications.

## Introduction

Plants recognize potential pathogens by perceiving elicitors commonly known as microbe-associated molecular patterns (MAMPs). Recognition of MAMPs then lead to induction of a series of defense signaling responses such as hypersensitive response (HR), production of reactive oxygen species (ROS) and various antimicrobial secondary metabolites. MAMPs cause oxidative burst through cell membrane bound NADPH oxidase during pathogen challenge and the ROS generated strengthens the cell wall by cross-linking glycoproteins or lipid peroxidation (Torres et al., 2006). ROS such as H_2_O_2_ being diffusible in nature across the cell membrane are also believed to act as intra or inter-cellular secondary messenger leading to expression of *NPR1*, a crucial mediator of systemic defense response (Mou et al., 2003). ROS as intracellular secondary messenger triggers a cascade of mitogen-activated protein kinases (MAPKs) (Jalmi and Sinha, 2015). Initially, the serine threonine kinase (STK) is activated which then phosphorylates the MAPKs as observed in the case of interactions between the hemi-biotroph pathogen *Pseudomonas syringae* pv. *tomato* and tomato (Petersen et al., 2000). Further, MAPK cascade is known to activate following perception of stimuli from pathogens by plant heterotrimeric G-protein leading to dissociation of Gα unit from the Gβγ unit (Su et al., 2015). However, the mechanisms are relatively explored for only necrotrophic and hemi-biotrophic pathogens but not for obligate biotrophs (Patel et al., 2016a). Not much is known about the defense responses generated during plant–fungal biotroph interactions particularly in relation to G-protein mediated signaling and specific roles of ROS, MAP kinases and the impact on lignifications.

Lignin either as complex polymers or other antimicrobial compounds produced in the lignin biosynthetic pathway has significant impact in resisting the invading pathogens. Structurally, lignins are three-dimensional polyphenols composed of three monolignols viz., *p*-coumaryl alcohols [*p*-hydroxyphenyl (H) lignin], coniferyl alcohols [guaiacyl (G) lignin], and sinapyl alcohols [syringyl (S) lignin] at various ratios. The secondary walls in gymnosperms mainly composed of lignins rich in guaiacyl (G) and deficient in syringyl (S) monolignols whereas lignins in dicot species are typically rich in both guaiacyl (G) and syringyl (S) monolignols. However, secondary walls of grass species is made up of *p*-hydroxyphenyl (H) monolignol and small amounts of guaiacyl (G) and syringyl (S) monolignols (Bonawitz and Chapple, 2010).

Monolignol biosynthesis occurs through conversion of the substrate phenylalanine followed by synthesis of common phenylpropanoid metabolites leading to the production of hydroxycinnamoyl-CoA esters. These esters further converts into different monolignols by branched pathways. Synthesis of these monolignols is governed primarily by about 10 enzymes via a complex cross connected pathway (Bonawitz and Chapple, 2010). Down regulation of any one of those enzyme producing genes can lead to strong reduction in lignin levels. Poor lignifications and stunted growth was observed in *Arabidopsis thaliana* and *Medicago sativa* after down regulation of the enzyme hydroxycinnamoyl CoA: shikimate hydroxycinnamoyl transferase (HCT) (Chen and Dixon, 2007; Shadle et al., 2007; Li et al., 2010). However, compared to HCT down regulation of other enzymes of the monolignol biosynthesis pathway have less effect on plant growth and development (Ranjeva et al., 1983; Chabannes et al., 2001a,b; Shadle et al., 2007; Jackson et al., 2008; Nakashima et al., 2008). In addition, knockdown of HCT in alfalfa increased SA content and transcript level of SA induced PR genes (Gallego-Giraldo et al., 2011) indicating possible enhancement in disease resistance without high lignifications. The results show a potential inverse relationship between levels of SA and lignifications. However, it is important to look into this aspect from disease resistance point of view in order to understand the host response toward a particular pathogen.

There are number of reports suggesting the role of beneficial rhizospheric microbes in activating the phenylpropanoid-mediated lignin biosynthesis (Singh et al., 2013; Srivastava et al., 2014). However, there is lack of clarity on the influence of beneficial rhizospheric microorganisms such as *Trichoderma* and fluorescent *Pseudomonas* on downstream signal transduction during host-powdery mildew interactions. In a previous study we demonstrated that during the interactions of pea with the powdery mildew pathogen *Erysiphe pisi*, transcript accumulation of Gα subunit was high whereas transcript accumulations of Gβ and Gγ were only basal (Patel et al., 2016a). The transcript accumulation pattern was distinct during the obligate fungal pathogen interaction with pea compared to other reports on necrotroph and hemi-biotroph interactions where activation of Gβ subunit was demonstrated (Suharsono et al., 2002; Llorente et al., 2005; Trusov et al., 2010). In order to understand the host responses during interaction with an obligate fungal pathogen we addressed the questions (i) whether NADPH oxidase is involved in generation of ROS during the interaction? (ii) whether the canonical MAP kinase cascade is involved in the interaction following ROS generation? and (iii) what is the pattern of lignifications during the host-pathogen interactions? We used pea and *E. pisi* as a model system to understand the issues. Further, we treated pea seeds with two rhizosphere compatible microbial strains *Pseudomonas fluorescens* and *Trichoderma asperellum* to understand how other external factors such as beneficial microbes influence the pea-*E. pisi* interaction. Information generated in the present study thus believed to strengthen our understanding on the interactions of plant with fungal biotrophic pathogens.

## Materials and Methods

### Experimental Setup

Two rhizospheric compatible bioagents *P. fluorescens* (OKC; GenBank accession JN128891) and *T. asperellum* (T42; GenBank accession JN128894) were used in the study (Patel et al., 2016a). A susceptible pea (*Pisum sativum*) cultivar AP3 was used as host of the obligate fungal pathogen *E. pisi*. Seeds of cv. AP3 were bioprimed with *Trichoderma* and *Pseudomonas* cells singly as well as in combination following the method reported by Yadav et al. (2013). *Pseudomonas* culture was inoculated in King’s B broth and incubated in incubator shaker at 27 ± 2°C for 24 h and then bacterial cells were harvested by centrifuging at 10000 rpm at 4°C. After harvesting, the cells were washed with distilled water (DW) three times and sterilized distilled water was added till the OD (optical density) 0.391 standardized for cell numbers 10^8^ ml^-1^. A 5 mm mycelia disk of *Trichoderma* was inoculated on PDA in Petri plates after 6 days when the plates were filled with green spores of the fungus. The spores were harvested by adding an adequate amount of sterilized DW and filtered through sterilized cotton to remove mycelia debris. DW was added further till OD is 1.141 standardized for spore numbers 10^8^ ml^-1^ of the suspension. Equal amount of both the spore suspensions were mixed for the co-inoculated treatment and CMC (carboxymethyl cellulose) was added @ 0.1% as sticker. Surface sterilized (0.1% HgCl_2_) pea seeds were added to the culture suspensions and incubated at room temperature for 4–6 h for soaking. Soaked seeds were sown in sterilized soil amended with 20% vermicompost. All the pots having treated and untreated seeds were kept in growth chamber at 21 ± 2°C and 16/8 h light/dark ratio. The powdery mildew pathogen *E. pisi*, which is an obligate fungal parasite was collected from agricultural farm of Banaras Hindu University and inoculated on the treated plants by dusting with the help of a fine brush. Pathogen inoculation was done after 21 days of plant growth as described by Singh et al. (2002).

### RNA Harvesting and Germ Tube Behavior

Pea leaves were collected after 24 and 48 h of pathogen inoculation for harvesting of RNA, studying the germ tube behavior on *E. pisi* conidia as well as for enzyme assays. Plant stem samples were also collected for histochemical staining for lignifications after 15 days of pathogen inoculation. Leaf samples were collected from both microbial treated and non-treated plants with or without pathogen challenge.

### Phenylalanine Ammonia Lyase (PAL) Assay

Leaf sample (0.5 g) from each treatment was taken and homogenized in 4 ml of borate buffer (pH 8.7; 4°C) and centrifuged at 13000 rpm for 15 min at 4°C. The supernatant was used as enzyme source. The reaction mixture contained 0.2 ml of enzyme extract, 1.3 ml distilled water and 1.0 ml of 0.1 M phenylalanine. The reaction mixture was incubated at 32°C for 30–60 min after the incubation and 0.5 ml of 1 M TCA (trichloro acetic acid) was added immediately to stop the reaction. OD optical density was measured at 290 nm by taking the control containing all the solutions except enzyme extract. Phenylalanine ammonia lyase (PAL) (EC 4.1.3.5) activity was also measured at 290 nm following formation of *trans*-cinnamic acid (Havir, 1987) and was expressed in terms of μmol l^-1^ TCA per g fresh weight (FW).

### Quantitative Estimation of H_2_O_2_

Leaf sample (0.1 g) from each of the treatments was grinded with help of mortar and pestles in ice bath in 2.0 ml of 0.1% (w/v) of TCA. The crushed material was centrifuged at 12,000 × *g* for 10 min and 0.5 ml of the supernatant was used for consequent steps. 10 mM potassium phosphate buffer (pH 7.0) and 1 ml of 1 M potassium iodide solution was added to supernatant and incubated at room temperature for 5 min. The oxidation product formed was measured spectrophotometrically at 390 nm (Velikova, 2000). The amount of H_2_O_2_ formed was determined by correlating with the standard curve made with known concentrations of H_2_O_2_ and expressed in nmol H_2_O_2_ g^-1^ FW.

### Histochemical Staining for Lignifications, Germ-Tube Behavior and Hypersensitive Response (HR)

Hand sectioned transverse sections (TSs) of pea stem from the first node were mounted in 1% phloroglucinol prepared in 95% ethanol, then transferred on glass slide and covered with cover-slip. Concentrated HCL was added near the edge of cover-slip so that HCL reach to the section (Guo et al., 2001). Pink color development showed lignin deposition. Observation was done under a compound light microscope (Nikon, Japan). For the germ tube behavior study leaf samples were destained in ethanol: acetic acid (3:1) solution. After leaf clearing leaves were stained with cotton blue and observed in a light compound microscope after mounting in glycerol. Germ tube lengths were calculated from 50 conidia per leaf from three different leaves of each treatment. For observation of HR, cleared leaves from different microbial treatments was observed under a light microscope after 72 h of pathogen challenge.

### Quantitative Estimation of Lignin

Quantitative estimation of lignin was done using the acid-solubilization method described by Dence (1992). Oven dry leaf and stem samples were crushed in mortar and pestle. 0.1 g of dry powdered sample was dissolved in 72% H_2_SO_4_. The acid added samples were incubated for 1 h at 30°C. 28 ml of sterilized distilled water was added after incubation. Further, autoclaving was done for 1 h at 121°C and 15 psi. Autoclaved samples were filtered in hot (80°C) condition by using Whatman No.1 filter paper. Filtrate was taken for spectrophotometric analysis at 205 nm. Results were described as lignin content in μg/g of dry weight.

### RNA Harvesting and cDNA Preparation

Total RNA was harvested from leaf samples after 24 h of pathogen inoculation by using the method described by Patel et al. (2016b). Amount of RNA was analyzed by NanoDrop 2000 (Thermo). Approximately, 3 μg total RNA was digested using RNase-free DNase I at 37°C to remove remaining genomic DNA. Purity of RNA was tested by agarose gel electrophoresis (1.2% agarose, at 75 V). cDNA was prepared by following the standard protocol (Sambrook and Russell, 2007) with the help of oligo (dT) primers, RNase inhibitor and reverse transcriptase enzyme. The prepared cDNAs of different treatments were quantified by using NanoDrop 2000 and diluted up to the concentration of 50 ng/μl.

### Quantitative Transcript Abundance Assay through qRT-PCR

Gene specific primers were designed by using gene sequences retrieved from the NCBI database and primer-3 software according to specifications described by Thornton and Basu (2011). The accession numbers of the selected genes are provided in Supplementary Table S1. The primers designed for targeted genes are listed in **Table 1**. qRT-PCR was carried out in iQ5 Real-Time PCR Detection System (Bio-Rad Laboratories, Munchen, Germany) by using Eva Green SYBR^®^ Green Supermix Kit (Biorad) and gene specific primers at the concentration of 0.1 μM. Transcript level of mRNA was normalized and determined with the level of ubiquitin. PCR conditions were adjusted according to the modified program described by Marone et al. (2001). Three technical replicates were used with the following conditions: denaturation at 95°C for 2 min, 40 repeats at 95°C for 20 s, 60°C for 30 s, and 72°C for 25 s. The data obtained by real time PCR of different treatments were normalized with CT value of ubiquitin using the 2^-ΔΔCT^ method (Schmittgen and Livak, 2008). The fold changes were obtained relative to control (C) treatment for each gene.

**Table 1:** List of gene specific forward (F) and reverse (R) primers used in the study.

S. No.	Gene name		Primer 5′–3′
1	C4H	F	CGGCTCCCGAACACGAAACG
		R	GCGGCTTCCGATTCCCAACC
2	COMT	F	TGATGTAGGTGGTGGTACTGGAGC
		R	TGCTTGCAAACATGTCTCCACCAA
3	F5H	F	ACGGAAACGGTAGCATCA
		R	TTGCTTCTTCCGCAGTTTC
4	HCT	F	TTTGGACTATCTTGAGCTACAACC
		R	CAACCAAAGTCAGCTTCATAGATT
5	PAL	F	ATGGTGTGAAGGTGGAGCTGTCA
		R	CGCCTTGTTTGGTTCGACGGT
6	CCoAOMT	F	GCCATGAAAGAGTTGAGAGAGG
		R	ATCAAGAACTGGAAGAGCTGGA
7	Laccase	F	CTGTGCCAGTGGGAGGA
		R	CAGCAGGTGGTGGAGGA
8	ABC transporters	F	GCACTGCAACTTGTTCTGTCCATT
		R	TCCTGCGCGATCATAAACTGTGA
9	NADPH oxidase	F	GGAGGAGCTTGGACACAGAAGC
		R	TCCACTTCCTCCACTCACCATCA
10	MAPK3	F	ACTCACGGTGGACAGTTCGTTCA
		R	CAGCAACCAACTCATTCGTCTCCG
11	MAPK6	F	TCTCCTCCAAGTACGCCCCG
		R	GGCAAGGATGTTCTCGTGGTCC
12	STK	F	GACCCAGGTGCTTCTCAGTCCA
		R	TCCACTCCATCTCCAAACTCACCC
13	PO	F	AACAAGGCCTCACCCCTACT
		R	GGCTAGGTTTGTTCCACTTCC
14	Ubiquitin	F	CCCCCAGACCAGCAAAGGTTGA
		R	TGTGTCTGAGCTCTCCACCTCCA

### Statistical Analysis

Statistical analysis was done by using SPSS version 16. Experiments were repeated twice using a completely randomized design. The data are expressed as the mean of three independent replications ± standard deviations. The treatment mean values were compared by Tukey’s test at *P* ≤ 0.05 significance level.

## Results

### NADPH Oxidase Activities, H_2_O_2_ Generation, HR, and Disease Development

**FIGURE 1:**
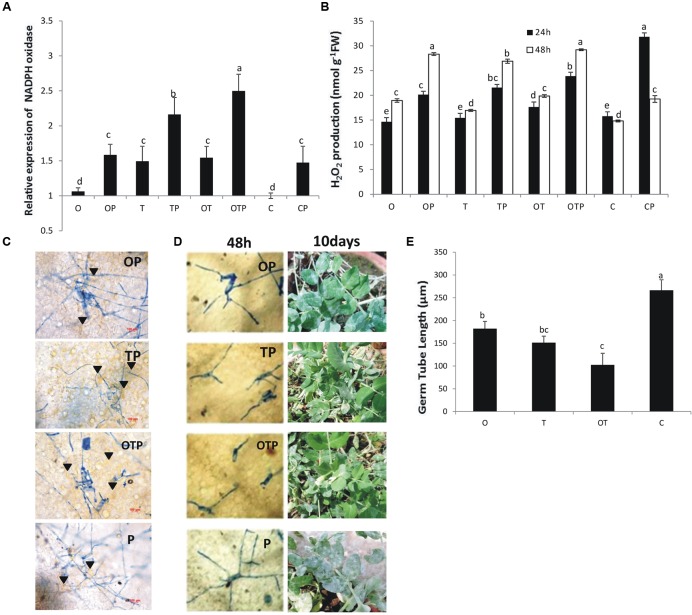
**Reactive oxygen species (ROS) generation and disease development in pea following inoculation with *Erysiphe pisi***. NADPH oxidase activity **(A)** after 48 h of pathogen inoculation which leads to production of ROS H_2_O_2_ **(B).** HR like responses **(C)** observed after 72 h of pathogen inoculation around the developing conidia (arrowhead). HR-like response was more in microbial treatments (OP, TP, and OTP) compared to non-microbial treatment (P). *E. pisi* development on pea leaves in different microbial treatments after 48 h and on intact plants after 10 days of pathogen inoculation **(D)**. Germ tube development of *E. pisi* on pea leaves in different treatments after 48 h **(E)**. Where O = *Pseudomonas fluorescens* (OKC), T = *Trichoderma asperellum* (T42), P = pathogen (*E. pisi*), C = control. Error bars in **(A,B,E)** represent SD (standard deviation) from means of three measurements. Different superscript letters indicate data significantly different from the other treatments (*P* ≤ 0.05; Tukey’s test).

Transcript accumulation pattern of NADPH oxidase (NOX) showed that its accumulation was highest (2.5 fold) in plants co-inoculated with the soil microbes and challenged with the pathogen *E. pisi* followed by pathogen challenged plants treated with only *Trichoderma* (2.25 fold) (**Figure 1A**). However, NOX accumulation was generally high in pathogen challenged plants treated with the soil microbes either singly or co-inoculated compared to non-challenged plants. H_2_O_2_ accumulation pattern also mimics the same pattern and it was observed that in pathogen challenged plants H_2_O_2_ accumulation was higher compared to pathogen non-challenged plants and highest accumulation being in the co-inoculated plants (**Figure 1B**). Further, accumulated levels of H_2_O_2_ were stable in pathogen-challenged and microbe inoculated plants at 48 h whereas in only pathogen-challenged plants without microbial treatments H_2_O_2_ levels declined significantly. HR development also correlated the observations of NOX and H_2_O_2_ and highest HR was observed in the co-inoculated plants (**Figure 1C**). Browning of cells was observed below the *E. pisi* colonies highest being in co-inoculated plants followed by *Trichoderma* inoculated plants and *Pseudomonas* inoculated plants. Least HR was observed in only pathogen challenged plants without microbial treatments. Germ tube development after 48 h (**Figure 1E**) and disease development after 10 days (**Figure 1D**) of pathogen inoculation were also least in co-inoculated plants and the disease development pattern was also in accordance to the pattern of NOX activities, H_2_O_2_ generation, and HR development.

### H_2_O_2_ Generation Does Not Lead to MAPK Activities

**FIGURE 2:**
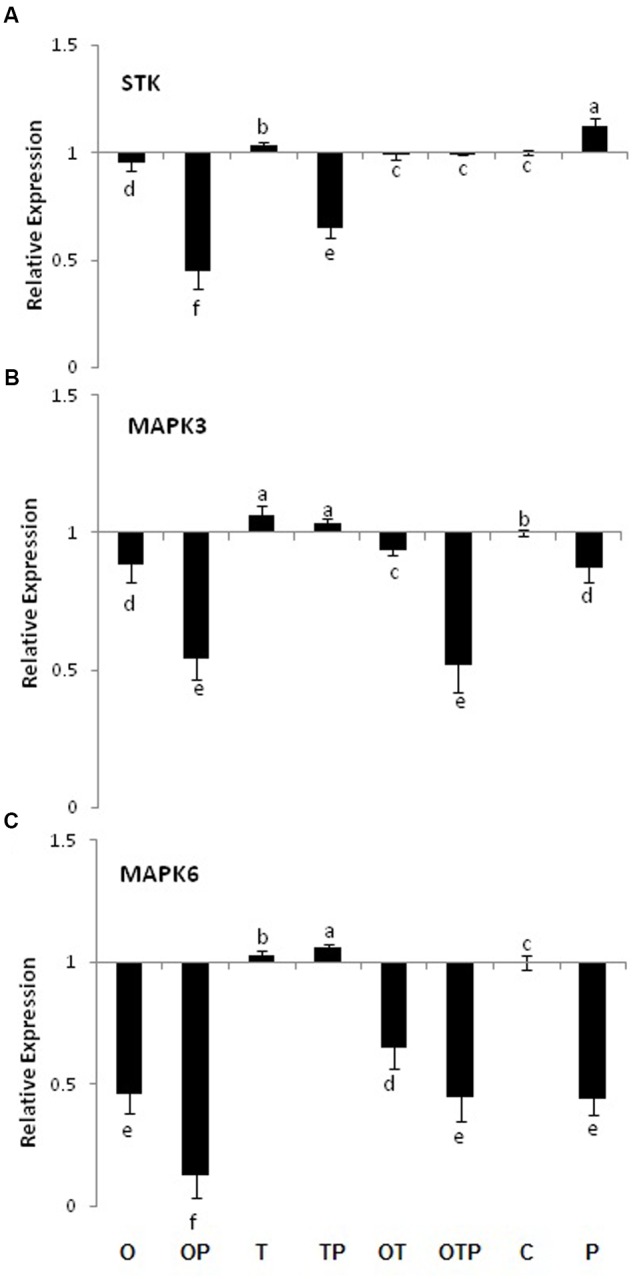
**Transcript accumulation pattern of MAP Kinases and their phosphorylating enzyme Serine/Threonine Kinase in pea leaves after 24 h of *E. pisi* inoculation. (A)** STK (Serine/threonine kinase), **(B)** MAP kinase 3 and **(C)** MAP kinase 6. Where O = *Pseudomonas fluorescens* (OKC), T = *Trichoderma asperellum* (T42), P = pathogen (*E. pisi*), C = control. Error bars represent SD from means of three measurements. Different superscript letters indicate data significantly different from the other treatments (*P* ≤ 0.05; Tukey’s test).

H_2_O_2_ generation is often linked with activation of the MAPK pathway. However, in the current study we did not see transcript accumulation of MAPKs and their phosphate donor STK (**Figure 2**). Rather, the transcript levels gone down several fold in most of the pathogen challenged plants. However, slightly increased level of MAPK3 and 6 transcripts were observed in only *Trichoderma* treated plants compared to several fold reduction in only *Pseudomonas* and co-inoculated plants. The results clearly demonstrated a negative correlation between H_2_O_2_ generation and MAPK activities.

### H_2_O_2_ Generation Positively Links to Phenylpropanoid Activation

**FIGURE 3:**
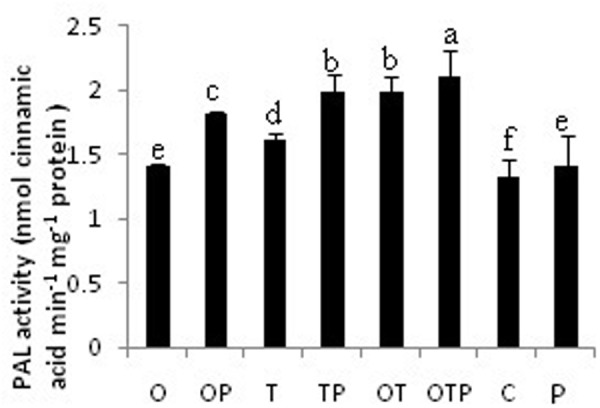
**Phenylpropanoid activities in pea leaves after 24 h of *E. pisi* inoculation**. Where O = *Pseudomonas fluorescens* (OKC), T = *Trichoderma asperellum* (T42), P = pathogen (*E. pisi*), C = control. Error bars represent SD from means of three measurements. Different superscript letters indicate data significantly different from the other treatments (*P* ≤ 0.05; Tukey’s test).

Phenylalanine ammonia lyase activity was increased in all microbial treatments but its activity was more in the pathogen challenged plants (**Figure 3**). Highest activity of PAL was observed in the co-inoculated plants challenged with the pathogen. Interestingly the co-inoculated plants without pathogen challenge also showed very high PAL activities and its activity is comparable with only *Trichoderma* treated plants and challenged with the pathogen. There was significant increase in PAL activity in only *Pseudomonas* treated plants also but its activity was low compared to only *Trichoderma* treated or co-inoculated plants. The pattern of PAL activities was also in accordance with the H_2_O_2_ accumulation pattern and therefore a positive correlation could be established between H_2_O_2_ generation and activation of PAL.

### Monolignol Synthesis and Lignifications

**FIGURE 4:**
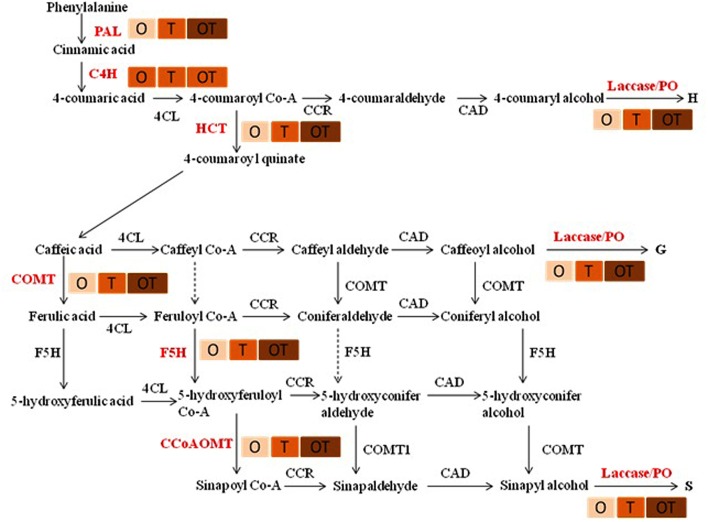
**Lignin monomer biosynthetic pathway demonstrating higher transcript accumulation of the key genes (shown in red colors)**. 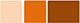 increase in color intensity showed level of expression of the genes in pea leaves.

**FIGURE 5:**
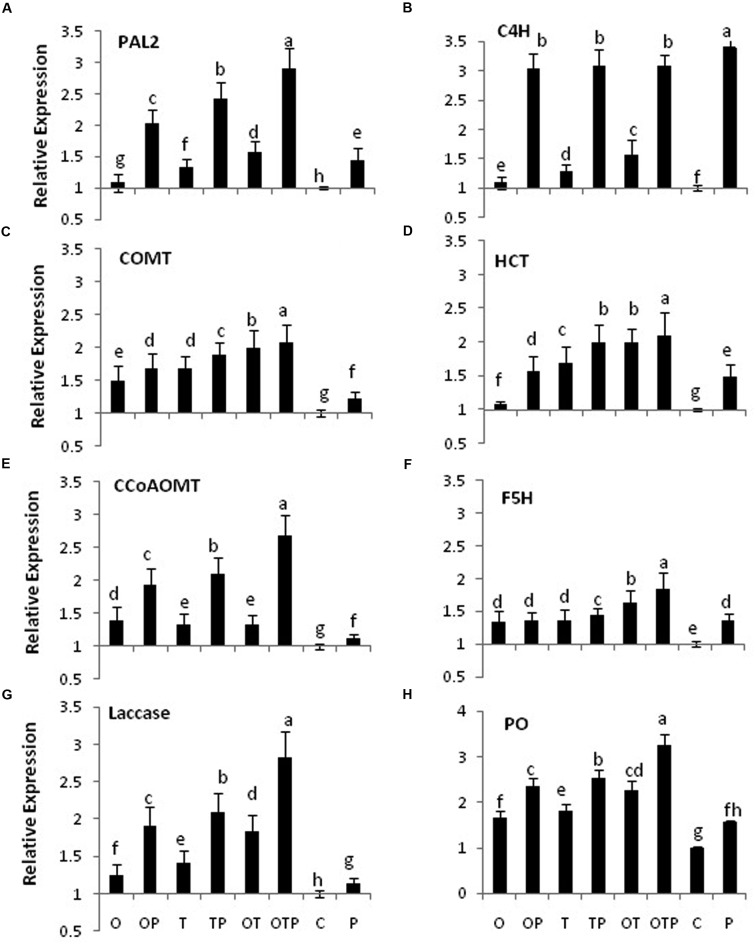
**Transcript accumulation pattern of some key genes involved in biosynthesis of lignin monomers after 24 h of *E. pisi* inoculation on pea leaves**. Transcript accumulation pattern of PAL (Phenylalanine ammonia lyase) **(A)**, C4H (Cinnamate 4-hydroxylase) **(B)**, COMT (Caffeic acid *O*-methyltransferase) **(C)**, HCT (Hydroxycinnamoyl CoA:shikimate hydroxycinnamoyl transferase) **(D)**, CCoAOMT (Caffeoyl CoA *O*-methyltransferase) **(E)**, F5H (Ferulate 5-hydroxylase) **(F)**, Laccase **(G)**, PO (Peroxidase) **(H)**. Where O = *Pseudomonas fluorescens* (OKC), T = *Trichoderma asperellum* (T42), P = pathogen (*E. pisi*), C = control. Error bars represent SD from means of three measurements. Different superscript letters indicate data significantly different from the other treatments (*P* ≤ 0.05; Tukey’s test).

**FIGURE 6:**
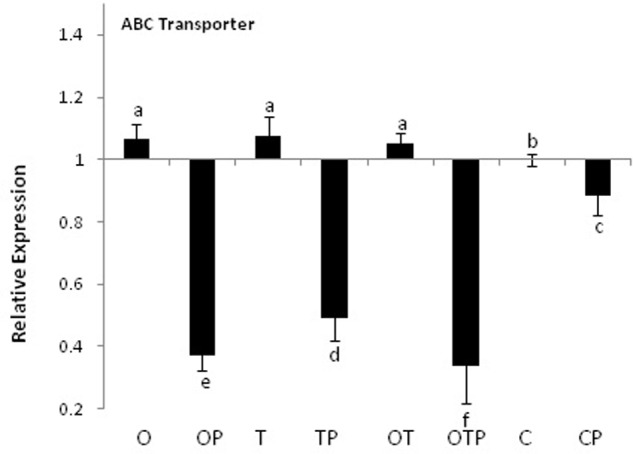
**Transcript accumulation pattern of the ABC transporter gene in pea leaves**. Where O = *Pseudomonas fluorescens* (OKC), T = *Trichoderma asperellum* (T42), P = pathogen (*E. pisi*), C = control. Error bars represent SD from means of three measurements. Different superscript letters indicate data significantly different from the other treatments (*P* ≤ 0.05; Tukey’s test).

Transcript accumulation patterns of key enzymes in the monolignol biosynthetic pathway revealed that the monolignol biosynthesis was enhanced (**Figure 4**). The transcript accumulation patterns in all the enzyme synthesizing genes were nearly similar and their highest accumulation was observed in the co-inoculated treatments challenged with the pathogen compared to the single microbial treatments (**Figure 5**). Transcript accumulation of the first enzyme producing gene of the phenylpropanoid pathway PAL that converts phenylalanine to cinnamic acid was observed higher in the microbe treated plants compared to untreated control and its expression increased further after pathogen challenge. Nearly two fold increase in PAL transcript accumulation was observed in co-inoculated plants challenged with the pathogen compared to the control plants (**Figure 5A**). Enzymatic estimation of PAL also showed a similar trend with the transcript accumulation pattern (**Figure 3**). When the expression of the next enzyme producing gene C4H (cinnamate 4-hydroxylase) was observed, that converts cinnamic acid to 4-coumeric acid, it also revealed that its transcript accumulation was high in all pathogen challenged microbial treatments although highest being in the pathogen challenged control plants (**Figure 5B**). Nearly two fold increases in its transcript accumulation was observed in all microbial treated plants challenged with the pathogen compared to control. Strong transcript accumulation of HCT (hydroxycinnamoyl CoA:shikimate hydroxycinnamoyl transferase) (**Figure 5C**), F5H (ferulate 5-hydroxylase) (**Figure 5D**), COMT (caffeic acid *O*-methyltransferase) (**Figure 5E**) and CCoAOMT (caffeoyl CoA *O*-methyltransferase) (**Figure 5F**) further indicated that precursors of all monolignols were produced in greater amounts (**Figure 4**). Interestingly, transcript accumulation of the ABC transporter associated with lignifications was down regulated in all pathogen challenged treatments (**Figure 6**). However, higher transcript accumulation of laccase (**Figure 5G**) and peroxidase (**Figure 5H**) confirm conversion of monolignol precursors to monolignols and cross-linking of monolignols into complex lignins.

**FIGURE 7:**
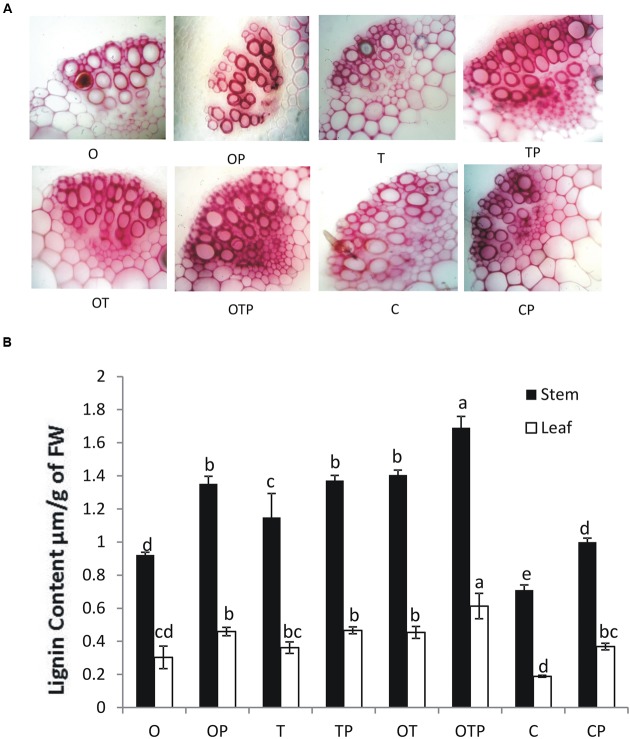
**(A)** Histochemical staining demonstrating lignin deposition in transverse sections of pea stem in different treatments after 15 days of *E. pisi* inoculation. Pink color represents lignin deposition. Intensity of color represents higher deposition of lignin. Where O = *Pseudomonas fluorescens* (OKC), T = *Trichoderma asperellum* (T42), P = pathogen (*E. pisi*), C = control. **(B)** Quantitative estimation of lignin in all treatments in leaf and stem. Error bars represent SD from means of three measurements. Different superscript letters indicate data significantly different from the other treatments in the same plant part (*P* ≤ 0.05; Tukey’s test).

Histochemical staining of stem sections revealed lignifications took place in higher amounts in the intercellular spaces in plants treated with the soil microbes particularly in greater amounts in the pathogen challenged plants (**Figure 7A**). Quantitative estimation of lignifications also revealed a similar pattern in both stem and leaf (**Figure 7B**). High transcript accumulation pattern of the key enzymes in the phenylpropanoid pathway (**Figure 5**) positively correlate activation of the phenylpropanoid pathway, synthesis of monolignols in higher amounts and cross-linking of the monolignols into complex lignin polymers in pea treated with the soil microbes particularly in the co-inoculated plants when challenged with the pathogen.

## Discussion

Plant disease resistance is a complex process and understanding the signaling processes is the key to understand host responses against an invading pathogen. Results from the present study involving interactions of pea with an obligate fungal pathogen *E. pisi* demonstrated that there is generation of ROS such as H_2_O_2_ in pea after pathogen challenge. H_2_O_2_ generation may be attributed to NADPH oxidase activities (Torres et al., 2006) and its activities were high in the present study in pathogen challenged pea leaves compared to non-challenged control. Interestingly, production of H_2_O_2_ was even higher when the rhizosphere microbes (OKC and T42) were co-inoculated. It was observed that H_2_O_2_ production was particularly high in treatments involving *Trichoderma* (T42) demonstrating commonalities between *T. asperellum* and *E. pisi* mediated production of ROS. In our previous study we demonstrated enhancement in ROS production by microbial co-inoculations (Patel et al., 2016a) but the role of NADPH oxidase is confirmed in the present study. A prominent role of NADPH oxidase in production of ROS in pathogen challenged plants has already been established in several cases (Grant et al., 2000; Torres and Dangl, 2005).

Activation of plasma membrane NADPH oxidase leads to ROS generation and activation of the phenylpropanoid pathway by activating the first enzyme of the phenylpropanoid pathway PAL (Hu et al., 2002). Similar observations were also made in the present study where activation of NADPH oxidase and ROS generation was followed by a significant induction of PAL activities particularly in the microbial treatments challenged with the obligate fungal pathogen *E. pisi*. Subsequent analysis of other genes of the phenylpropanoid pathway revealed high transcript accumulation of most of the important genes of the phenylpropanoid pathway namely C4H, HCT, COMT, CCoAOMT, and F5H. High transcript accumulation of HCT strongly indicates probability of synthesis of G and S monolignols along with H monolignols. Accumulation of significantly high CCoAOMT transcripts is seen as a possibility that between the two monolignols S monolignol accumulation may be high as the probability of synthesis of its precursor Sinapoyl CoA is high due to high CCoAOMT activities. Similar results were also demonstrated in grape plants challenged with *Botrytis cinerea* (Ali et al., 2011) and in Chinese cabbage after inoculation with the pathogen *Erwinia carotovora* ssp. *carotovora* (Zhang et al., 2007). However, down regulation of the ABC transporter, a homolog of *Arabidopsis ABCB15* that positively correlates with lignifications (Kaneda et al., 2011), in all pathogen challenged treatments showed non-participation of the ABC transporter during lignifications. Down regulation of the ABC transporter thus theoretically rules out active transport of monolignols and higher lignifications observed may be due to passive transport of the monolignols to the apoplast (Liu, 2012). In addition, high transcript accumulations of laccase and peroxidase further strengthens the possibilities of oxidative polymerization of monolignols by the two enzymes to form lignin macromolecules in the cell wall. Their transcript accumulations were particularly high in microbial co-inoculated treatment which indicated synergistic effect of the co-inoculated microbes during pathogen challenge. Significance of compatible rhizosphere microbial mixtures in eliciting the phenylpropanoid activities was also reported earlier (Jain et al., 2012; Sarma et al., 2015). However, in most cases they were tested against necrotrophic pathogens and the present results demonstrated the effect of an obligate fungal pathogen on lignifications.

In our previous report we showed that during pea and *E. pisi* interactions there is significantly high transcript accumulation of Gα subunit of the pea heterotrimeric G protein and transcript accumulations of Gβ and Gγ were only basal (Patel et al., 2016a). Activation of Gβ subunit is associated with MAP kinase mediated signaling (Cheng et al., 2015). However, Gα mediated downstream signaling events are not clearly understood. In the present study also we did not find any evidence of activation of MAP kinase pathway. Neither the MAPK homologs MAPK3/MAPK6 known for participation in defense signaling (Petersen et al., 2000; Asai et al., 2002) nor their phosphorylating enzyme STK (Krusell et al., 2002) was found active during the interaction. The results thus strengthen our previous observation where we reported non-activation of the Gβ subunit. Further it can also be said that the downstream signaling of Gα subunit might not follow the MAP kinase pathway. It was also interesting to note that neither the pathogen nor the introduced rhizosphere microbes stimulate transcript accumulation of the MAP kinase homologs. Rather they were down regulated in all pathogen treatments. Although there was slight increase in STK transcript accumulation in only pathogen treatment, introduction of the rhizosphere microbes caused down regulation of the same.

Development of *E. pisi* on pea was least in the co-inoculated treatment in the present study. Similar reports were made by Fernando et al. (2007), and they showed that the mixture of *Pseudomonas chlororaphis* PA-23 and *Bacillus amyloliquefaciens* BS6 significantly reducing the stem rot on canola petals caused by *Sclerotinia sclerotiorum* under field conditions by inhibiting ascospore germination and by inducing plant defense related enzymes. Less disease development in the present study may be attributed to higher activation of NADPH oxidase activities leading to generation of ROS such as H_2_O_2_ that served as a signaling molecule for activation of the phenylpropanoid pathway. The effect was enhanced further by co-inoculation of the two rhizosphere compatible microbes *T. asperellum* and *P. fluorescens*. However, it remains to explore how the heterotrimeric G-proteins mediated signaling takes place following infection by an obligate fungal pathogen. The current understanding is only that in contrast to Gβ mediated signaling in necrotrophs, it is the Gα mediated signaling that takes place following challenge by an obligate fungal pathogen. The line of evidence from the current study also points out that the MAP kinase cascade that is activated during defense responses via Gβ mediation in infection by necrotrophs does not appear to be activated during Gα mediated responses in pea during challenge with the obligate fungal pathogen *E. pisi.* From the results of the current study it can be inferred that rhizosphere microbes *T. asperellum* (T42) and *P. fluorescens* (OKC) enhances *E. pisi* resistance in pea by enhancing ROS generation and lignifications.

## Author Contributions

BS and HS conceived the idea and planned the experiments. JP conducted the experiments. JP, BS, and RK interpreted and analyzed the results. BS, JP, and RU written the manuscript.

## Conflict of Interest Statement

The authors declare that the research was conducted in the absence of any commercial or financial relationships that could be construed as a potential conflict of interest.
