# Supplementary material for: Trichoderma asperellum (T42) and Pseudomonas fluorescens (OKC)-Enhances Resistance of Pea against Erysiphe pisi through Enhanced ROS Generation and Lignifications
Source: Front Microbiol. 2017 Mar 2;8:306. doi: 10.3389/fmicb.2017.00306 (PMC5332396; doi:10.3389/fmicb.2017.00306)
Supplement: Supplementary file 1 [file Table_1.DOCX]

**Supplementary Table 1. Accession IDs of CDS of genes whose sequences were used in the study for designing primers.**

| Sl. No. | Gene Name | Accession numbers |
| --- | --- | --- |
| 1 | **C4H** | >gi\|9957080\|gb\|AF175275.1\| *Pisum sativum* trans-cinnamic acid hydroxylase (CYP73A9) gene, CYP73A9-v1 allele, partial cds. |
| 2 | **COMT** | >gi\|357464228:105-1202 *Medicago truncatula* Caffeic acid O-methyltransferase (MTR_3g092900) mRNA, complete cds  >gi\|571460397:45-1145 PREDICTED: *Glycine max* caffeic acid 3-O-methyltransferase-like (LOC100777636), mRNA  >gi\|502136646:38-1135 PREDICTED: *Cicer arietinum* caffeic acid 3-O-methyltransferase-like (LOC101489645), mRNA |
| 3 | **F5H** | >gi\|397790695\|gb\|JN714252.1\| *Medicago sativa* subsp. *caerulea* isolate PI 314267A ferulate 5-hydroxylase (F5H) gene, exon 2 and partial cds  >gi\|397790697\|gb\|JN714253.1\| *Medicago sativa* subsp. *caerulea* isolate PI 464723A ferulate 5-hydroxylase (F5H) gene, exon 2 and partial cds  >gi\|346229108\|gb\|HQ636604.1\| *Glycine max* coniferylaldehyde 5-hydroxylase mRNA, complete cds |
| 4 | **HCT** | >gi\|358346438\|ref\|XM_003637227.1\| *Medicago truncatula* Hydroxycinnamoyl CoA shikimate/quinatehydroxycinnamoyltransferase (MTR_080s0019) mRNA, complete cds.  >gi\|356550672:212-1531 PREDICTED: *Glycine max* hydroxycinnamoyl-Coenzyme A shikimate/quinatehydroxycinnamoyltransferase-like (LOC100801923), mRNA  >gi\|356557206\|ref\|XM_003546861.1\| PREDICTED: *Glycine max* hydroxycinnamoyl-Coenzyme A shikimate/quinatehydroxycinnamoyltransferase-like (LOC100805843), mRNA  >gi\|502148122:141-1451 PREDICTED: *Cicer arietinum* shikimate O-hydroxycinnamoyltransferase-like (LOC101501844), transcript variant X2, mRNA  >gi\|502148120:14-1363 PREDICTED: *Cicer arietinum* shikimate O-hydroxycinnamoyltransferase-like (LOC101501844), transcript variant X1, mRNA |
| 5 | **PAL2** | >(gi\|217983:2085-2512, 2603-4349) *Pisum sativum* PAL2 gene for phenylalanine ammonia-lyase, complete cds  >(gi\|217981:1509-1933, 2486-4232) *Pisum sativum* PAL1 gene for phenylalanine ammonia-lyase, complete cds |
| 6 | **CCoAoMT** | >gi\|357475282\|ref\|XM_003607879.1\| *Medicago truncatula* Caffeoyl-CoA O-methyltransferase (MTR_4g085590) mRNA, complete cds  >gi\|502143167\|ref\|XM_004505185.1\| PREDICTED: *Cicer arietinum* caffeoyl-CoA O-methyltransferase-like (LOC101508155), mRNA  >gi\|356499754\|ref\|XM_003518654.1\| PREDICTED: *Glycine max* caffeoyl-CoA O-methyltransferase-like, transcript variant 2 (LOC100811247), mRNA |
| 7 | **Laccase** | >gb\|DQ335245.1\|:115-1830 *Pisum sativum* putative copper ion-binding laccase mRNA, complete cds |
| 8 | **ABC transporters** | >(gi\|657371793:5304612-5304648, 5304881-5306310) *Medicago truncatula* strain A17 chromosome 8, whole genome shotgun sequence  >NM_113754.3:268-3990 Arabidopsis thaliana ABC transporter family protein (*ABCB15*), mRNA |
| 9 | **NADPH Oxidase** | >gi\|357504428:1-1098 *Medicago truncatula* NADPH oxidase (MTR_7g038480) mRNA, complete cds  >gi\|33286865\|gb\|AF405422.2\| *Pisum sativum* ferric-chelate reductase, complete cds  >gi\|502157094:77-2227 PREDICTED: *Cicer arietinum* ferric reduction oxidase 2-like (LOC101499003), mRNA  >gi\|571465181:12-2132 PREDICTED: *Glycine max* ferric reduction oxidase 2-like (LOC100790114), transcript variant 2, mRNA |
| 10 | **MAPK3** | >gb\|AF153061.1\|:64-1179 *Pisum sativum* MAP kinase 3 (Mapk3) mRNA, complete cds |
| 11 | **MAPK6** | >(gi\|330250293:18138477-18138709, 18138931-18139060, 18139510-18139647, 18139739-18140071, 18140152-18140332, 18140521-18140693) *Arabidopsis thaliana*  >gi\|573960723:1-1092 PREDICTED: *Oryza brachyantha* mitogen-activated protein kinase 6-like (LOC102701584), partial mRNA  >gi\|145360962:171-1358 *Arabidopsis thaliana* MAP kinase 6 mRNA, complete cds |
| 12 | **STK** | >(gi\|24940243:86-2657, 2767-3125) *Pisum sativum* sym29 gene for serine-threonine protein kinase, exons 1-2 |
| 13 | **PO** | >gi\|37051106:1-818 *Pisum sativum* PRX mRNA for peroxidase, partial cds  >gi\|62909962:55-1107 *Pisum sativum* mRNA for peroxidase, complete cds, clone:PsPOX29 |
| 14 | **Ubiquitin** | >[XM_003627103.1](http://www.ncbi.nlm.nih.gov/entrez/viewer.fcgi?db=nucleotide&id=357513724) *Medicago truncatula* Ubiquitin (MTR_8g018230) mRNA, complete cds |
